# Nicotinamide Increases Intracellular NAD^+^ Content to Enhance Autophagy-Mediated Group A Streptococcal Clearance in Endothelial Cells

**DOI:** 10.3389/fmicb.2020.00117

**Published:** 2020-02-11

**Authors:** Cheng-Lu Hsieh, Shu-Ying Hsieh, Hsuan-Min Huang, Shiou-Ling Lu, Hiroko Omori, Po-Xing Zheng, Yen-Ning Ho, Yi-Lin Cheng, Yee-Shin Lin, Chuan Chiang-Ni, Pei-Jane Tsai, Shu-Ying Wang, Ching-Chuan Liu, Takeshi Noda, Jiunn-Jong Wu

**Affiliations:** ^1^Institute of Basic Medical Sciences, College of Medicine, National Cheng Kung University, Tainan, Taiwan; ^2^Institute of Molecular Medicine, College of Medicine, National Cheng Kung University, Tainan, Taiwan; ^3^Department of Medical Laboratory Science and Biotechnology, College of Medicine, National Cheng Kung University, Tainan, Taiwan; ^4^Center for Frontier Oral Science, Graduate School of Dentistry, Osaka University, Osaka, Japan; ^5^Research Institute for Microbial Diseases, Osaka University, Osaka, Japan; ^6^Center of Infectious Disease and Signaling Research, College of Medicine, National Cheng Kung University, Tainan, Taiwan; ^7^Department of Biotechnology and Laboratory Science in Medicine, School of Biomedical Science and Engineering, National Yang-Ming University, Taipei, Taiwan; ^8^Department of Microbiology and Immunology, College of Medicine, National Cheng Kung University, Tainan, Taiwan; ^9^Department of Microbiology & Immunology, College of Medicine, Chang Gung University, Taoyuan, Taiwan; ^10^Molecular Infectious Disease Research Center, Chang Gung Memorial Hospital, Taoyuan, Taiwan; ^11^Department of Pediatrics, College of Medicine, National Cheng Kung University and Hospital, Tainan, Taiwan

**Keywords:** Group A streptococcus (GAS), nicotinamide (NAM), NAD^+^ homeostasis, intracellular survival, endothelial cells (ECs)

## Abstract

Group A streptococcus (GAS) is a versatile pathogen that causes a wide spectrum of diseases in humans. Invading host cells is a known strategy for GAS to avoid antibiotic killing and immune recognition. However, the underlying mechanisms of GAS resistance to intracellular killing need to be explored. Endothelial HMEC-1 cells were infected with GAS, methicillin-resistant *Staphylococcus aureus* (MRSA) and *Salmonella* Typhimurium under nicotinamide (NAM)-supplemented conditions. The intracellular NAD^+^ level and cell viability were respectively measured by NAD^+^ quantification kit and protease-based cytotoxicity assay. Moreover, the intracellular bacteria were analyzed by colony-forming assay, transmission electron microscopy, and confocal microscopy. We found that supplementation with exogenous nicotinamide during infection significantly inhibited the growth of intracellular GAS in endothelial cells. Moreover, the NAD^+^ content and NAD^+^/NADH ratio of GAS-infected endothelial cells were dramatically increased, whereas the cell cytotoxicity was decreased by exogenous nicotinamide treatment. After knockdown of the autophagy-related ATG9A, the intracellular bacterial load was increased in nicotinamide-treated endothelial cells. The results of Western blot and transmission electron microscopy also revealed that cells treated with nicotinamide can increase autophagy-associated LC3 conversion and double-membrane formation during GAS infection. Confocal microscopy images further showed that more GAS-containing vacuoles were colocalized with lysosome under nicotinamide-supplemented conditions than without nicotinamide treatment. In contrast to GAS, supplementation with exogenous nicotinamide did not effectively inhibit the growth of MRSA or *S.* Typhimurium in endothelial cells. These results indicate that intracellular NAD^+^ homeostasis is crucial for controlling intracellular GAS infection in endothelial cells. In addition, nicotinamide may be a potential new therapeutic agent to overcome persistent infections of GAS.

## Introduction

Group A streptococcus (GAS) is recognized as one of the major human pathogens that not only provokes mild skin and throat infections such as impetigo or pharyngitis, but also occasionally causes serious infections such as streptococcal toxic shock syndrome and necrotizing fasciitis (Cunningham, 2008; Walker et al., 2014). Although antibiotics are effective for treating GAS infections, accumulating evidence has shown that invading host cells could be one of the strategies used by GAS to avoid killing by host immune responses and antibiotics, which may contribute to persistent infection seen in 5–30% of individuals (Osterlund et al., 1997; Marouni et al., 2004; Kaplan et al., 2006).

Nicotinamide adenine dinucleotide (NAD^+^) and its reduced form (NADH) have been known as important cofactors that are coupled to the redox reactions for energy production, or used as enzymatic cofactors involving in hundreds of biochemical functions within living cells (Canto et al., 2015). Nicotinamide (NAM) is a vitamin B3 derivative that has been used as a metabolic precursor of NAD^+^ to raise intracellular NAD^+^ level for improvement of metabolic diseases involving energy production (Fricker et al., 2018). Autophagy, the cellular degradation of ubiquitinated proteins in lysosomes under stress conditions, is one of the most tightly regulated homeostatic processes involving intracellular NAD^+^ (Klionsky et al., 2016; Kocaturk and Gozuacik, 2018). Moreover, autophagy also plays a crucial role in innate immune defenses against invading pathogens including *Listeria monocytogenes*, *Salmonella* Typhimurium, and GAS (Castrejón-Jiménez et al., 2015; Kimmey and Stallings, 2016; Bah and Vergne, 2017). In order to survive in host cells, GAS expresses various virulence factors to impair autophagic clearance, including streptococcal cysteine protease SpeB, streptolysin O (SLO), and NAD-glycohydrolase (NADase) (Sakurai et al., 2010; Barnett et al., 2013; Mestre and Colombo, 2013; O’Seaghdha and Wessels, 2013; O’Neill et al., 2016; Sharma et al., 2016). NADase is a potent hydrolase involved in the consumption of NAD^+^ that leads to intracellular energy collapse and programmed necrosis of infected cells (Chandrasekaran and Caparon, 2015, 2016; Pajuelo et al., 2018). In addition, several studies have indicated that NADase is involved with the structural and functional stabilization of SLO, which contributes to enhance GAS pathogenesis and global dissemination of serotype M1 and M89 GAS, indicating that NADase plays an important role during GAS infection (Michos et al., 2006; Turner et al., 2015; Zhu et al., 2015; Velarde et al., 2017; Barnett et al., 2018). However, the mechanisms of NAD^+^ homeostasis controlling GAS survival in the host are complicated and need to be explored.

Previously, we have found that defective acidification of autophagosomes allows GAS growth in endothelial cells (Lu et al., 2015). NADase is responsible for the depletion of intracellular NAD^+^ and inhibition of autophagosomal acidification, which results in the multiplication of GAS in endothelial cells (Hsieh et al., 2018). In this study, we demonstrate that supplementation with exogenous NAM significantly restores the intracellular NAD^+^ content and NAD^+^/NADH ratio, which enhances the acidification of GAS-containing autophagosomes and clearance of intracellular GAS within endothelial cells.

## Materials and Methods

### Cell Culture

Human microvascular endothelial cell line-1 (HMEC-1) cells were cultured in endothelial growth medium M200 with low serum growth factors (Gibco Life Technologies, Grand Island, NY, United States) and 10% fetal bovine serum (FBS) at 37°C in a humidified incubator with 5% CO_2_. When the cell confluence reached 80%, cells were detached with trypsin-EDTA (Gibco Life Technologies) and seeded at the density of 0.75 × 10^6^ cells/dish in 10-cm dishes for maintenance or 3 × 10^5^ cells/well in 6-well plates for the intracellular bacteria survival assay and confocal microscopy.

### Bacteria and Cultural Conditions

Group A streptococcus strains SF370 (M1 serotype) and NZ131 (M49 serotype) were purchased from the American Type Culture Collection (Manassas, VA, United States). GAS strain A20 (M1 serotype) was isolated from the blood of a patient with necrotizing fasciitis (Zheng et al., 2013). Methicillin-resistant *Staphylococcus aureus* (MRSA) and *Salmonella* Typhimurium were isolated from patients with bacteremia. All strains were susceptible to gentamicin and cultured on tryptic soy agar containing 5% defibrinated sheep blood or tryptic soy broth (Becton Dickinson, Sparks, MD, United States) supplemented with 0.5% yeast extract (TSBY).

### Intracellular Bacterial Survival Assay

The cell infection was described in the previous study with modifications (Hsieh et al., 2018). In brief, the overnight bacterial cultures were transferred and grown to the exponential growth phase, and then resuspended in endothelial growth medium M200. HMEC-1 cells were seeded in 6-well plates at a density of 3 × 10^5^ cells/well and infected with bacteria at different multiplicities of infection (M.O.I.) to ensure equivalent intracellular bacterial load at 1 h of infection. Plates were then centrifuged at 500 *g* for 5 min to synchronize the infection and incubated in humidified 5% CO_2_ at 37°C for 30 min. After infection, the infected cells were treated with gentamicin (125 μg/mL) to kill extracellular bacteria. Subsequently, cells were maintained in M200 medium with or without β-NAD, NADH, or NAM for additional 2 and 4 h. At indicated times post infection, cells were lysed by deionized water and plated on TSBY agar plates to evaluate the intracellular GAS growth within endothelial cells.

### Determination of NADase Activity

The NADase activity in GAS culture supernatants was measured by the qualitative fluorescence assay according to the method described above by Bricker et al. (2002). Briefly, the bacterial supernatants from different culture treatments were harvested by centrifugation at 2,330 *g* for 10 min. The supernatants were reacted with 1 mM of β-NAD (Sigma-Aldrich, St. Louis, MO, United States) at 37°C in 5% CO_2_ for 1 h. After incubation, sodium hydroxide (PanReac AppliChem, Barcelona, Spain) was added to develop reactions at room temperature in the dark for 1 h. The fluorescence intensity of β-NAD was determined by Tecan M200 Pro Infinite plate reader (Tecan, Crailsheim, Germany) at excitation wavelength 360 nm. The NADase activity of each sample was expressed as a relative percentage compared with uninoculated culture medium.

### Quantification of Intracellular NAD^+^ Level

The change of intracellular NAD^+^ content was determined by the NAD^+^/NADH quantification kit (Sigma-Aldrich), according to the manufacturer’s instructions. Briefly, a total of 2 × 10^5^ cells were harvested by trypsinization and suspended in NAD^+^/NADH extraction buffer to lyse the cells by freezing and thawing three times. The 10-kDa cut-off spin column (GE Healthcare, Buckinghamshire, United Kingdom) was used to remove NADH degrading enzymes by centrifugation at 21,000 *g*, 4°C for 60 min. The intracellular NAD^+^ of each sample was decomposed in 60°C for 30 min and then transferred to microtiter plates to determine the concentration of intracellular NADH. In addition, total intracellular NADH of each sample was reacted with NAD cycling enzyme to convert NAD^+^ to NADH. Subsequently, all samples were loaded into microtiter plates and mixed with NADH developer and incubated at room temperature for 1 h. After reaction, the absorbance of each sample was measured by ELISA reader (Tecan, infinite M200) at the wavelength of 450 nm. The NAD^+^/NADH ratio was calculated to evaluate the metabolic status of cells following a formula: [(NADH_*total*_−NADH)/NADH].

### Cell Cytotoxicity Assay

To determine cell cytotoxicity of GAS-infected cells under NAM treatment, the activity of dead-cell protease was measured by CytoTox-Glo cytotoxicity assay kit according to the manufacturer’s instructions (Promega, Madison, WI, United States). Briefly, cell were infected with GAS as described above. The cell culture supernatants were harvested at indicated times post infection, and co-incubated with luminogenic substrate at room temperature for 15 min. The luminescent intensity was measured by Tecan M200 Pro Infinite plate reader (Tecan). The cell cytotoxicity of each sample was expressed as a relative percentage compared with non-infected cells.

### RNA Interference

The expression of ATG9A in endothelial HMEC-1 cells was downregulated through lentiviral expression of a short hairpin RNA (clone 81, TRCN0000244081 containing target sequence 5′-AGTCACCTTGGCACCATATTG-3′; clone 82, TRCN0000244082 containing target sequence 5′-GTGGACTATGACATCCTATTT -3′; clone 83, TRCN0000244083 containing target sequence 5′-TGTAGGAGCAGGATGGAAATA-3′). The shRNA lentiviral clones were obtained from the National RNAi Core Facility at the Academia Sinica in Taiwan. Endothelial HMEC-1 cells were seeded in 6-well plates at a density of 2 × 10^5^ cells/well and infected with lentivirus at a M.O.I of 3 in M200 medium containing 8 μg/mL of polybrene at 37°C in 5% CO_2_ for 24 h. After transduction, cells were treated with 5 μg/mL of puromycin (Sigma-Aldrich) for three generations to select the stable clone. The efficiency of ATG9A knockdown was evaluated by Western blot analysis.

### Transmission Electron Microscopy (TEM)

Endothelial HMEC-1 cells were infected with GAS at M.O.I. of 1 for 30 min, and treated with gentamicin to kill extracellular bacteria. After treatment, cells were maintained in NAM-supplemented medium for additional 2 h. Cells were detached by trypsinization and gently resuspended in phenol red-free medium containing 5% FBS and 40% Dextran T2000. Cells were kept on ice and cryo-fixed in a high-pressure freezer (Leica EM HPM100) according to manufacturer’s instructions. After fixation, samples were dehydrated by freeze substitution and embedded in plastic (Epon812, TAAB Laboratories Equipment, Aldermaston, United Kingdom). The embedded samples were cut into 70 nm ultrathin sections, and stained with saturated uranyl acetate and Reynolds lead citrate solution. TEM images were acquired with a JEOL JEM-1011 transmission electron microscope (JEOL, JEM-1011, Tokyo, Japan).

### Western Blotting

Cells were infected with GAS as described above. At indicated times post infection, cells were lysed with RIPA lysis buffer containing protease inhibitor (Promega). Cell lysates were quantified using Bradford protein assay (Bio-Rad, Hercules, CA, United States) and boiled in SDS sample buffer for 10 min. All samples were then separated by 12% SDS-polyacrylamide gel and transferred onto the polyvinylidene difluoride (PVDF) membranes (Millipore, Boston, MA, United States). After blocking with 5% skim milk, the membranes were incubated with primary antibody against ATG9A (Abcam Technology, Cambridge, MA, United States), LC3 (MBL, Nagoya, Japan), or β-actin (novusbio, Littleton, CO) in blocking buffer at 4°C for overnight. After incubation, horseradish peroxidase (HRP)-conjugated secondary antibody (Jackson Immunoresearch Laboratories, West Grove, PA, United States) were used to visualize the Western blot. All images were acquired with the ImageQuant LAS-4000 imaging system (GE Healthcare Life Sciences, Pittsburgh, PA, United States).

### Confocal Microscopy

The confocal microscopy was performed as previously described (Hsieh et al., 2018). All images were acquired and analyzed with the confocal microscope LSM 880 (Carl Zeiss, Jena, Germany). The percentage of colocalization between GAS, autophagosomal LC3 (MBL), lysosomal marker LAMP1 (Cell Signaling Technology, Danvers, MA, United States), and acidotropic probe Lysotracker (Invitrogen Molecular Probes, Eugene, OR, United States) was quantified from five independent fields of each experiment.

### Statistical Analysis

Data are representative of three independent experiments and expressed as the mean ± standard error of the mean (SEM). GraphPad Prism 5.0 was used to plot all graphs and evaluate statistical significance by 1-way or 2-way ANOVA with *post hoc* Tukey’s or Bonferroni multiple comparison test, respectively. Results were considered as statistically significant when the *p*-value was less than 0.05, and marked by an asterisk (^∗^) in figures.

## Results

### Nicotinamide Inhibits Intracellular GAS Growth in Endothelial Cells

**FIGURE 1:**
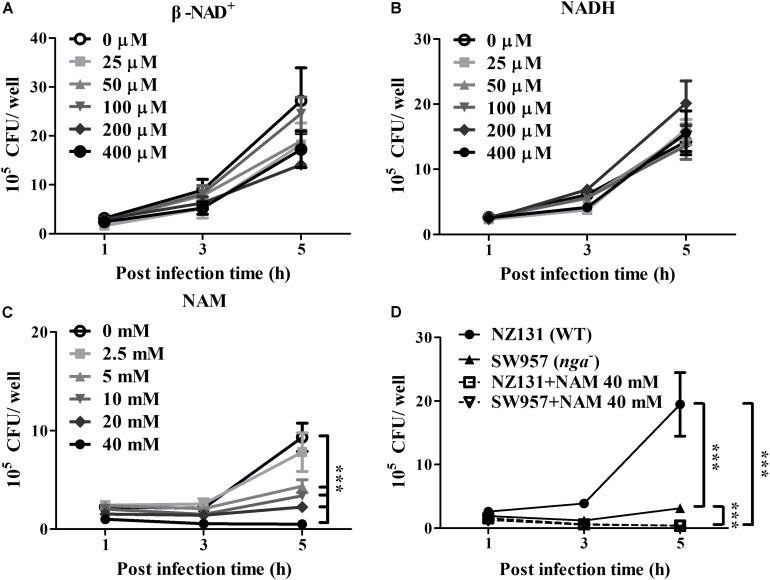
Nicotinamide inhibits intracellular GAS growth in endothelial cells. The intracellular growth of GAS was analyzed in endothelial HMEC-1 cells under exogenous NAD^+^ substrate supplementation. HMEC-1 cells were infected with wild-type NZ131 at M.O.I. of 1 and gentamicin was used to kill extracellular bacteria. Cells subsequently were maintained in the fresh M200 medium with/without different concentrations of **(A)** β-NAD, **(B)** NADH, or **(C)** nicotinamide (NAM) for an additional 2 and 4 h. The intracellular bacterial load was counted by CFU-based assay. **(D)** The intracellular growth of GAS was analyzed in the NAM-treated endothelial HMEC-1 cells. HMEC-1 cells were infected with wild-type NZ131 or NADase-knockout strain SW957, treated with gentamicin, and maintained in 40 mM of NAM. The intracellular bacterial load was counted by CFU-based assay. The data represent the means ± SEM of at least three independent experiments. ***p < 0.001 (2-way ANOVA).

NAD-glycohydrolase is a potent glycohydrolase that cleaves β-NAD to produce nicotinamide and ADP-ribose, leading to disrupted energy homeostasis and promoting bacterial survival in host cells (Bastiat-Sempe et al., 2014; Hsieh et al., 2018; Pajuelo et al., 2018). However, the underlying mechanisms associating NAD^+^ metabolism with intracellular bacterial survival are complicated and poorly understood. To clarify whether NAD^+^ depletion or accumulated metabolic products contributed to GAS survival in endothelial cells, exogenous β-NAD, NADH and nicotinamide (NAM) were supplemented in culture medium, and the GAS survival in endothelial HMEC-1 cells was evaluated by a colony-forming assay. The results revealed no significant difference in the bacterial count of intracellular GAS within endothelial HMEC-1 cells under different concentrations of β-NAD^+^ and NADH after 5 h of infection (Figures 1A,B). In contrast, supplementation with 2.5 to 40 mM of NAM was able to inhibit the intracellular GAS growth within endothelial HMEC-1 cells in a time and dose-dependent manner (Figure 1C).

Nicotinamide has been extensively used to treat various clinical disorders and skin infections (Rolfe, 2014). To investigate whether NAM directly inhibits bacterial survival, the TSBY broth was supplemented with NAM to measure the growth of GAS *in vitro*. The results showed that the bacterial growth was not affected in the presence of 2.5 to 40 mM of NAM, when compared with the control medium (Supplementary Figure S1A). Previous reports showed that the enzymatic activity of NADase is required for GAS survival in host cells (O’Seaghdha and Wessels, 2013; Sharma et al., 2016). To explore whether NAM could mediate intracellular GAS growth through repressing NADase activity, the GAS was cultured to mid-logarithmic phase in TSBY broth supplemented with different concentrations of NAM. The results showed that deletion of *nga* (SW957) caused loss of the NADase activity, compared to wild-type NZ131. Moreover, NADase activity of wild-type NZ131 in GAS culture supernatant was decreased to 45 and 60.8% in the presence of 40 mM NAM at 4 and 6 h of incubation, respectively (Supplementary Figure S1B), when compared with untreated group. In order to clarify whether NAM reduced NADase activity to mediate intracellular GAS survival, the NADase-encoding gene (*nga*) was deleted by homologous recombination (Hsieh et al., 2018). The endothelial HMEC-1 cells were then infected with *nga* mutant under NAM treatment. The results showed that the intracellular growth of the NADase mutant was significant decreased when compared with the wild-type NZ131 in non-NAM-treated HMEC-1 cells. Moreover, supplementation with exogenous NAM further reduced both wild-type NZ131 and NADase mutant growth in HMEC-1 cells during infection (Figure 1D). These results imply that NADase activity contributes to intracellular GAS survival, but NAM may trigger other mechanisms to inhibit intracellular GAS growth in endothelial cells.

### Nicotinamide Elevates Intracellular NAD^+^ Content and the NAD^+^/NADH Ratio of Endothelial Cells

**FIGURE 2:**
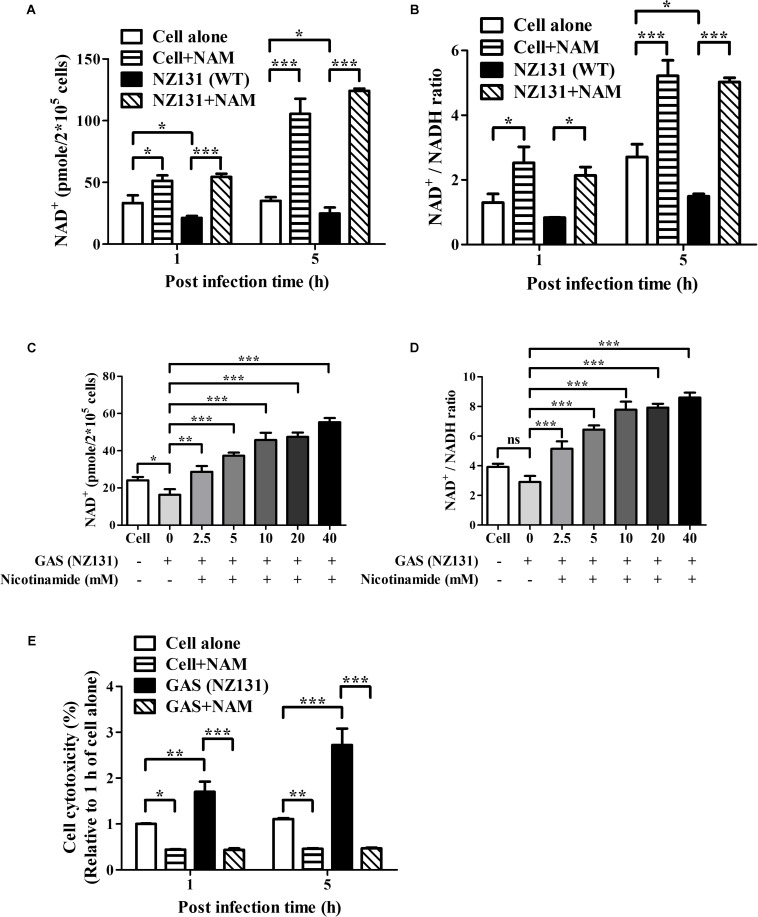
Nicotinamide increases intracellular NAD^+^ content and NAD^+^/NADH ratio of endothelial cells. **(A,C)** The intracellular NAD^+^ content of endothelial HMEC-1 cells was measured after nicotinamide (NAM) treatment. The cell lysates were extracted from GAS-infected HMEC-1 cells with/without NAM treatment and analyzed by a NAD^+^/NADH quantification kit. **(B,D)** The NAD^+^/NADH ratio of GAS-infected HMEC-1 cells was calculated following a formula: [(NADH_*total*_ – NADH)/NADH]. **(E)** The cell viability of GAS-infected cells after NAM treatment were measured by CytoTox-Glo cytotoxicity assay kit. The cell culture supernatants were harvested after GAS infection with/without NAM treatment at indicated time point, and then co-incubated with luminogenic substrate. The cell cytotoxicity was expressed as relative percentage compared to 1 h of cell alone. The data represent the means ± SEM of at least three independent experiments. *p < 0.05; **p < 0.01; ***p < 0.001 (1- or 2-way ANOVA).

Nicotinamide can be used as a precursor for the biosynthesis of NAD^+^ through the salvage pathway in mammalian cells (Canto et al., 2015). We were interested to understand whether NAM could increase the intracellular NAD^+^ content in endothelial cells. NAM was supplemented in culture medium, and then intracellular NAD^+^ and NAD^+^/NADH ratio were evaluated at indicated time points post infection. As expected, the results revealed that NZ131-infected cells have lower NAD^+^ content and NAD^+^/NADH ratios than non-infected cells at 1 and 5 h of infection. In contrast, supplementation with exogenous NAM could significantly increase intracellular NAD^+^ content and the NAD^+^/NADH ratio in endothelial cells at 1 and 5 h of infection, compared with untreated cells (Figures 2A,B). In order to illustrate the correlation between exogenous NAM supplementation and intracellular NAD^+^ content in endothelial cells during GAS infection, the intracellular NAD^+^ content and NAD^+^/NADH ratio of HMEC-1 cells were analyzed by treatment with different concentrations of NAM (2.5 to 40 mM). The results showed that supplementation with exogenous NAM can gradually elevate intracellular NAD^+^ content and the NAD^+^/NADH ratio in NZ131-infected HMEC-1 cells (Figures 2C,D), suggesting that both intracellular NAD^+^ level and NAD^+^/NADH ratio are correlated with NAM treatment in endothelial cells during GAS infection. In addition to NAM, the intracellular NAD^+^ content of NZ131-infected cells was also measured after exogenous β-NAD and NADH supplementation. The results showed that the intracellular NAD^+^ content was not increased after β-NAD and NADH treatment, when compared with untreated and NAM-treated cells at 5 h of infection (Supplementary Figure S2).

Nicotinamide adenine dinucleotide (NAD^+^) is considered as a critical molecule involved in diverse physiological functions including energy production and cell viability (Filomeni et al., 2015). To illustrate whether increased NAD^+^ contributed to cell survival, the protease-based cytotoxicity assay was used to measure the cell viability of GAS-infected cells under NAM treatment. The results showed that the GAS infection induced higher cytotoxicity than non-infected cells, but NAM treatment can dramatically decrease cytotoxicity in both non-infected and GAS-infected cells at 1 and 5 h of infection (Figure 2E).

### Nicotinamide Modulates Autophagic Activation to Inhibit GAS Growth in Endothelial Cells

**FIGURE 3:**
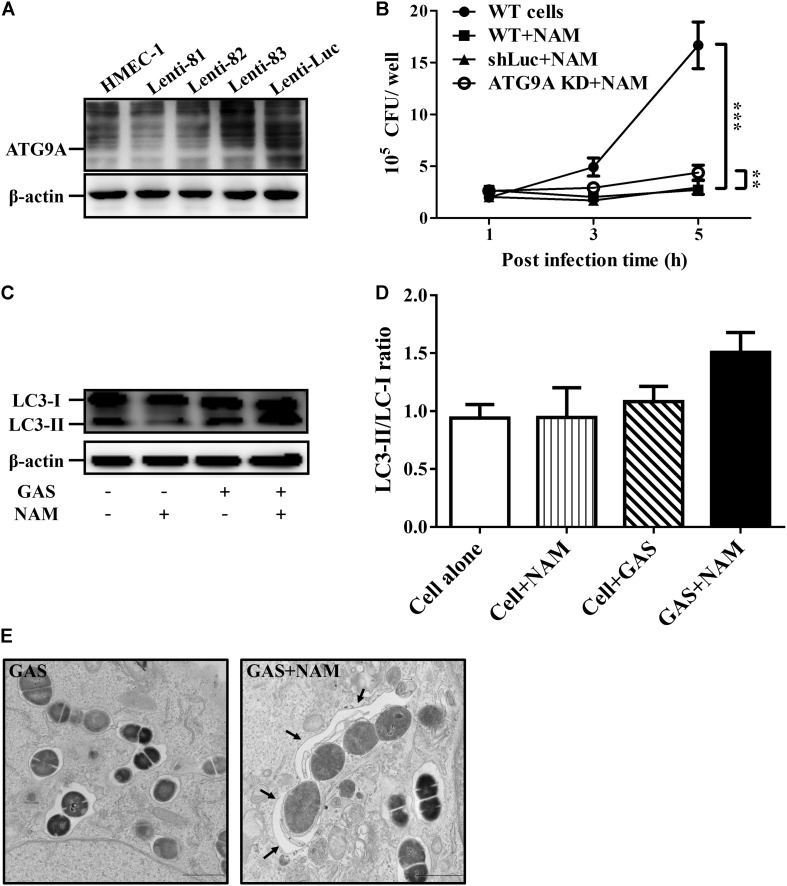
Nicotinamide activates autophagy to inhibit intracellular GAS growth in endothelial cells. **(A)** The expression of ATG9A in endothelial HMEC-1 cells. HMEC-1 cells were transfected with three lentivirus-based shRNAs (shATG9A 81, 82, and 83) to silence the ATG9A expression or Luciferase shRNA (shLuc) for a negative control. The expression of ATG9A was examined by Western blot analysis. **(B)** The intracellular growth of GAS was evaluated in ATG9A-knockdown (KD) HMEC-1 cells under nicotinamide (NAM) treatment. The wild-type and lentivirus-infected HMEC-1 cells (shLuc and clone 82) were infected with GAS at M.O.I. of 1, and gentamicin was used to kill extracellular bacteria. The intracellular bacterial load was counted by CFU-based assays. The data represent the means ± SEM of at least three independent experiments. **p < 0.01; ***p < 0.001 (2-way ANOVA). **(C)** The LC3 conversion of GAS-infected HMEC-1 cells under NAM treatment were analyzed by Western blotting. **(D)** The relative band intensities of LC3-I and LC3-II were measured by densitometry analysis using ImageJ software and expressed as the ratio of LC3-II/LC3-I. **(E)** Cells were infected with GAS and treated with/without NAM at 1 h of infection. The membrane structure of GAS-containing vacuoles were observed by conventional transmission electron microscopy (TEM). Black arrow indicate the double membrane compartment. Scale bar = 1 μm.

Several studies have indicated that NAD^+^ homeostasis plays a prominent role in the regulation of autophagy responses (Preyat and Leo, 2016; Zhang et al., 2016). Accordingly, the autophagy-related ATG9A was knocked down by lentiviral short hairpin RNA (shRNA) to explore whether the increasing NAD^+^ was associated with autophagy-mediated intracellular GAS clearance in endothelial cells. The results of Western blot analysis showed that the clone 82 had the highest knockdown efficiency of ATG9A, compared to wild-type and negative control shLuc-infeced HMEC-1 cells (Figure 3A). In addition, the autophagy-related LC3-II form was decreased in ATG9A-knockdown cells when compared to wild-type cells (Supplementary Figure S3A). The clone 82 was subsequently chosen to infect with GAS for analyzing bacterial intracellular growth under NAM treatment. The results showed that the intracellular bacterial load was significantly increased in ATG9A-knockdown HMEC-1 cells under 40 mM of NAM supplementation, when compared to wild-type and shLuc-infected cells (Figure 3B). In order to clarify whether autophagy could be activated by NAM treatment, the LC3 conversion was analyzed in endothelial HMEC-1 cells treated with NAM during GAS infection. The results of the Western blotting showed that a trend of increasing level of LC3-II form was observed in NAM-treated cells at 5 h of infection, however, it has no significance when compared to non-treated cells (lane 3 versus lane 4, *p*-value = 0.1, Figures 3C,D). In contrast, the LC3-II form was not induced in ATG9A-knockdown HMEC-1 cells after NAM treatment (Supplementary Figure S3B). Next, transmission electron microscopy (TEM) was further used to visualize the autophagosome membrane structure in GAS-infected HMEC-1 cells. The images revealed that intracellular GAS was surrounded by the single membrane structure, but double membrane-engulfed GAS was markedly observed in NAM-treated HMEC-1 cells (Figure 3E). Theses evidences suggest that intracellular GAS can be sequestered by autophagy in endothelial cells after NAM treatment.

### Nicotinamide Enhances Intracellular Trafficking of GAS-Containing Vacuoles to Lysosome and Acidification in Endothelial Cells

**FIGURE 4:**
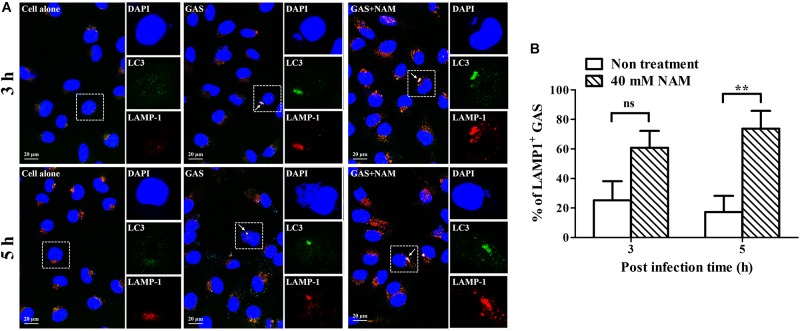
Nicotinamide enhances GAS-containing vacuole trafficking to lysosomes in endothelial cells. **(A)** The colocalization of intracellular GAS with autophagosomes and lysosomes was analyzed in nicotinamide (NAM)-treated endothelial HMEC-1 cells. Confocal microscopy was used to observe the colocalization of GAS (DAPI, blue) with LC3 (Alexa 488, green) and LAMP-1 (Alexa 594, red) in HMEC-1 cells under NAM treatment at 3 and 5 h of infection. Representative images are shown from three independent experiments. Scale bar = 20 μm. **(B)** The intracellular bacteria associated with LAMP-1 at 3 and 5 h of infection were quantified from confocal microscopy images. At least 100 intracellular GAS were quantified for each time point in at least three independent experiments. The data represent the means ± SEM of at least three independent experiments. ns, not significant; **p < 0.01 (2-way ANOVA).

Previous studies have shown that GAS can survive in host cells through avoidance of lysosomal degradation with bacteria-containing vacuoles (O’Seaghdha and Wessels, 2013; Sharma et al., 2016; Hsieh et al., 2018). Therefore, whether NAM could enhance lysosomal recruitment with GAS-containing vacuoles to inhibit GAS growth in endothelial cells was further analyzed. The intracellular localization of bacteria, LC3 decorated vesicles, and lysosomes in endothelial HMEC-1 cells were observed by confocal microscopy after NAM treatment. Confocal images showed that the intracellular bacteria were colocalized with LC3-positive vacuoles, but the colocalization of intracellular bacteria with LC3 puncta was reduced in HMEC-1 cells without NAM treatment after 5 h of infection. In contrast, more intracellular bacteria were colocalized with LC3-positive vacuoles in NAM-treated HMEC-1 cells compared to without NAM treatment after 5 h of infection (Figure 4A). Furthermore, the membrane glycoprotein LAMP-1 was used as a marker for visualizing the colocalization of lysosomes with intracellular GAS in endothelial HMEC-1 cells. The results showed that only 25 and 17% of intracellular GAS was associated with LAMP-1 in untreated cells at 3 and 5 h of infection, respectively. Under the NAM treatment, 61 and 74% of intracellular GAS was colocalized with LAMP-1 at 3 and 5 h of infection, respectively (Figures 4A,B).

**FIGURE 5:**
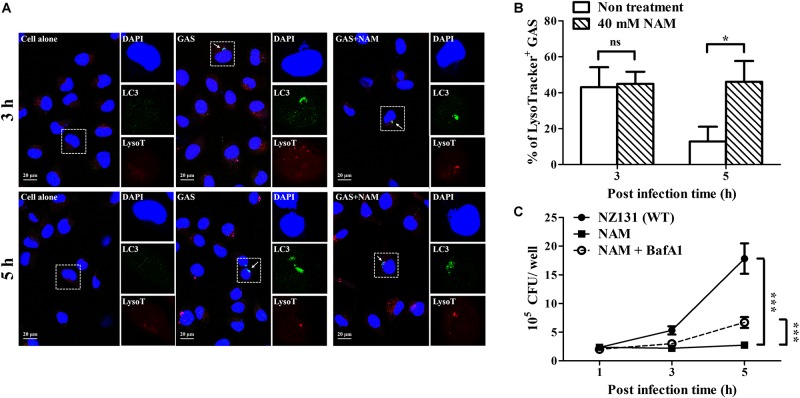
Nicotinamide increases acidification of GAS-containing vacuole in endothelial cells. **(A)** The colocalization of intracellular GAS with acidified autophagosomes was analyzed in nicotinamide (NAM)-treated HMEC-1 cells. The intracellular localization of GAS (DAPI, blue), LC3 (Alexa 488, green), and acidification (acidotropic indicator LysoTracker Red DND-99, red) in endothelial cells was observed by confocal microscopy at 3 and 5 h of infection. Representative images are shown from three independent experiments. Scale bar = 20 μm. **(B)** The percentage of intracellular bacteria associated with LysoTracker was quantified at 3 and 5 h of infection. At least 100 intracellular GAS were quantified for each time point in at least three independent experiments. **(C)** The intracellular growth of GAS was analyzed in endothelial HMEC-1 cells under bafilomycin A1 (BafA1) and NAM cotreatment. HMEC-1 cells with/without pretreatment with 200 nM BafA1, were then infected with GAS at M.O.I. of 1 in presence or absence of 40 mM NAM. The intracellular bacterial load was counted by CFU-based assays. The data represent the means ± SEM of at least three independent experiments. ns, not significant; *p < 0.05; ***p < 0.001 (2-way ANOVA).

Sufficient acidification is required for lysosomal enzyme activity to eliminate intracellular bacteria in endothelial cells and macrophages (Bastiat-Sempe et al., 2014; Valderrama and Nizet, 2018). In order to clarify whether the GAS-containing vacuoles were successfully acidified after NAM treatment, the fluorescent indicator Lysotracker was used to observe the acidification within NAM-treated endothelial HMEC-1 cells during GAS infection. The results revealed that about 40% of intracellular bacteria were colocalized with Lysotracker under NAM-treated and untreated conditions at 3 h of infection. However, the colocalization of intracellular GAS with Lysotracker was higher in NAM-treated endothelial HMEC-1 cells compared to cells without NAM treatments at 5 h of infection (51 vs. 13%, respectively) (Figures 5A,B). The activity of lysosomal H^+^-ATPase was inhibited by bafilomycin A1 to evaluate the roles of acidification for intracellular survival of GAS in NAM-treated endothelial HMEC-1 cells. The results showed that the intracellular bacterial load was significantly increased following treatment with bafilomycin A1 at 5 h of infection compared to untreated cells under NAM-supplemented conditions (Figure 5C). These results indicate that treatment with exogenous NAM markedly enhances lysosome-mediated acidification to inhibit intracellular GAS growth in endothelial cells.

### Nicotinamide Specifically Inhibits Multiplication of Intracellular GAS in Endothelial Cells

**FIGURE 6:**
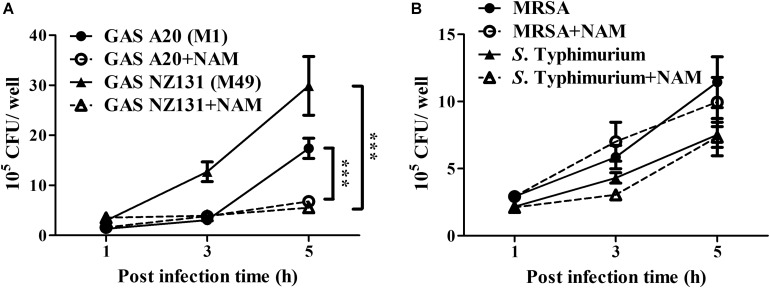
Nicotinamide specifically inhibits intracellular multiplication of GAS in endothelial cells. The intracellular growth of GAS, MRSA, and *S.* Typhimurium was analyzed in nicotinamide (NAM)-treated endothelial HMEC-1 cells. HMEC-1 cells were infected with **(A)** serotype M1 A20 at M.O.I. of 10, M49 NZ131 at M.O.I of 1 or **(B)** MRSA and *S.* Typhimurium at M.O.I of 1. The extracellular bacteria were killed by gentamicin and intracellular bacterial load was counted by CFU-based assays. The data represent the means ± SEM of at least three independent experiments. ***p < 0.001 (2-way ANOVA).

Nicotinamide and its derivatives have been extensively used to treat bacterial and viral infections (Murray, 2003; Singhal and Cheng, 2019). Based on these findings, we sought to examine whether NAM could inhibit other pathogens’ survival in endothelial cells. The serotype M1 and M49 GAS, MRSA, and *S.* Typhimurium were utilized to infect endothelial HMEC-1 cells and the intracellular bacterial growth was measured by a colony-forming assay. The results showed that the intracellular growth of both serotype M1 (strain A20) and M49 (strain NZ131) was significantly reduced in NAM-treated endothelial HMEC-1 cells at 5 h of infection, compared to non-treated cells (Figure 6A). However, supplementation with exogenous NAM did not significantly inhibit the growth of MRSA and *S.* Typhimurium in endothelial HMEC-1 cells (Figure 6B), implying that these pathogens may use different mechanisms to escape NAM-mediated clearance in endothelial cells.

## Discussion

**FIGURE 7:**
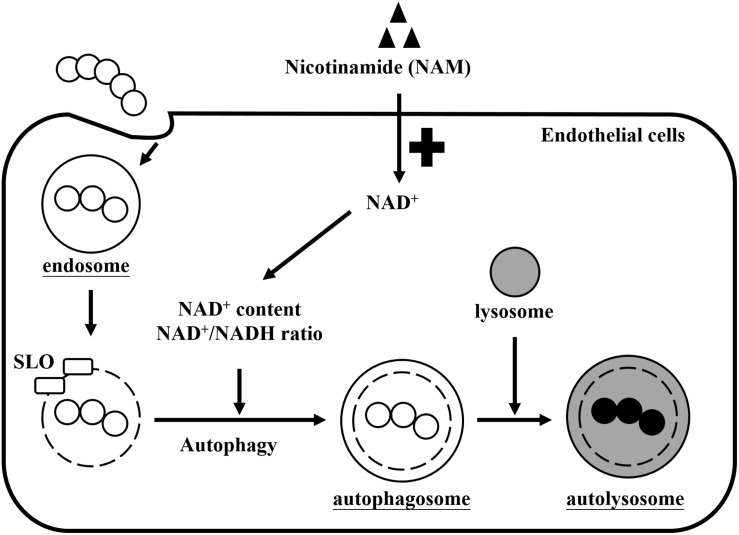
Effects of nicotinamide on GAS survival in endothelial cells. GAS is internalized into cells through endocytosis to form the endosomes. However, SLO is expressed to damage the endosomal membrane and further induces autophagy responses. GAS depletes intracellular NAD^+^ promoting their intracellular survival, but cells supplemented with exogenous nicotinamide (NAM) can restore the intracellular NAD^+^ content and NAD^+^/NADH ratio, resulting in GAS clearance by autophagy in endothelial cells.

Nicotinamide adenine dinucleotide (NAD^+^) is one of the most important molecules involved in energy generation and signal transduction to control cellular metabolism in live cells (Canto et al., 2015). However, several studies found that many pathogens express multiple toxins to influence intracellular NAD^+^ homeostasis of infected cells, which results in energy collapse, impaired host defense, immunopathology, and increased pathogen survival (Bastiat-Sempe et al., 2014; Sun et al., 2015; Bhat et al., 2016; Mesquita et al., 2016; Pajuelo et al., 2018). In this study, we found that supplementation with exogenous NAM could increase intracellular NAD^+^ levels and the NAD^+^/NADH ratio that contributed to enhance intracellular GAS clearance by autophagy in endothelial cells (Figure 7).

In order to clarify the correlation between the intracellular NAD^+^ content and bacterial survival of infected cells, the exogenous NAD^+^-associated substrates were supplemented in GAS-infected endothelial cells and bacterial survival was analyzed by a colony-formation assay. Our results demonstrated that supplementation with exogenous NAM not only dramatically increased intracellular NAD^+^ level and NAD^+^/NADH ratio, but also inhibited GAS growth in endothelial cells in a dose- and time-dependent manner (Figures 1, 2). In contrast to NAM, the intracellular NAD^+^ content and bacterial load were not affected by the exogenous β-NAD and NADH treatments (Supplementary Figure S2), which might be due to the membrane impermeability (Canto et al., 2015). In addition to NAM, intracellular NAD^+^ is also generated from tryptophan through a *de novo* biosynthetic pathway in the cells (Moffett and Namboodiri, 2003). Recent studies have shown that tryptophan catabolism plays a crucial role against bacterial and viral infections, including those caused by *Clostridium difficile*, *Mycobacterium tuberculosis*, and hepatitis B and C viruses (Larrea et al., 2007; El-Zaatari et al., 2014; Schmidt and Schultze, 2014). However, whether tryptophan is involved in NAD^+^ fluctuation influencing GAS survival in endothelial cells needs to be evaluated. These results indicate that the importance of intracellular NAD^+^ homeostasis on pathogens’ survival in the host.

Our results have shown that the given exogenous NAM can increase the conversion of LC3-I to LC3-II and induce autophagic double membrane formation to inhibit GAS survival in endothelial cells (Figures 3–5). The LC3-II level and ratio of LC3-II/LC3-I have been considered as a reliable marker for reflecting the activation of autophagy (Kadowaki and Karim, 2009). Recently, several studies have shown that supplementation with NAM can induce the conversion of LC3-I to LC3-II in chronic hypoxic myocardial cells, which support our results that the LC3-II was increased after NAM treatment in GAS-infected endothelial cells (Kang and Hwang, 2009; Hwang and Song, 2017; Li et al., 2019). These evidences indicate that NAM can increase autophagic activation under different stress conditions. However, the LC3-II-decorated single-membrane phagosomes or vacuoles (LAP) recently has been found in microbial survival within phagocytic and non-phagocytotic cells (Schille et al., 2018; Upadhyay and Philips, 2019). Therefore, the signaling pathways and membrane structure can be utilized to define the difference between the conventional autophagosome and LC3-associated phagosome (LAPosome) (Martinez et al., 2015; Schille et al., 2018). Autophagy is a tightly regulated process influenced by many NAD^+^-consuming enzymes including ADP-ribose polymerase-1 (PARP-1) and sirtuins (Zhang et al., 2016). Several studies have demonstrated that the NAD^+^-dependent sirtuins are involved in bacterial survival by regulating autophagy via activation of the AMPK/mTOR signaling pathway in intestinal epithelial cells and macrophages (Ganesan et al., 2017; Al Azzaz et al., 2018; Li et al., 2019; Yang et al., 2019). Moreover, exogenous NAM treatment can enhance Sirtuin-1 activity to control autophagy-mediated mitochondrial quality in human fibroblasts (Kang and Hwang, 2009; Jang et al., 2012; Shen et al., 2017). On the other hand, reactive oxygen species (ROS) are considered as another critical mediators of NAD^+^ in autophagy responses (Filomeni et al., 2015; Preyat and Leo, 2016). We recently found that GAS infection can induce ROS production to stimulate single membrane LAPosome formation for bacterial survival and activate the inhibition of mTOR for GAS escaping from autophagic clearance in endothelial cells. In contrast, suppression of ROS level significantly enhance GAS clearance through converting defective LAPosome to conventional double membrane autophagosome in endothelial cells (Lu et al., 2017; Cheng et al., 2019). Moreover, autophagy could be upregulated by supplementation with exogenous NAD^+^, which can suppress PARP-1-mediated necrotic death of retinal pigment epithelium cells under oxidative stress (Zhu et al., 2016). These evidences suggest that NAM may modulate sirtuins activity or ROS production to regulate signaling transduction of conventional autophagy for intracellular GAS clearance in endothelial cells, but the underlying mechanisms require further investigation.

Nicotinamide has been considered as a safe and readily available therapeutic agent to improve the metabolic diseases, neurodegeneration, and skin infection through raising intracellular NAD^+^ level (Knip et al., 2000; Fricker et al., 2018). In this study, we showed that supplementation with exogenous NAM greatly reduced the intracellular growth of both serotype M1 and M49 GAS in endothelial cells, but did not dramatically inhibit the growth of MRSA and *S.* Typhimurium (Figure 6). Recent studies have shown that the *M. tuberculosis* depletes intracellular NAD^+^ content to enhance their intracellular survival, but treatment with NAM can restore intracellular NAD^+^ level and reduce the bacterial load of *M. tuberculosis* in macrophages (Sun et al., 2015; Pajuelo et al., 2018). Interestingly, NADase depleting intracellular NAD^+^ is required for the intracellular survival of GAS and *M. tuberculosis* in host cells, but the NADase activity of MRSA and *S*. Typhimurium cannot be determined in the culture supernatants (data not shown). These results imply that GAS and *M. tuberculosis* may share similar NAD^+^-associated mechanisms for intracellular survival in host cells.

In summary, we conclude that intracellular GAS survival within endothelial cells is linked to NAD^+^ homeostasis, indicating that NAM and its analogs may provide novel therapeutic strategies against GAS infection.

## Data Availability Statement

All datasets generated for this study are included in the article/Supplementary Material.

## Author Contributions

Y-SL, CC-N, P-JT, S-YW, C-CL, TN, and J-JW conceived and designed the study. C-LH, S-YH, H-MH, Y-NH, P-XZ, and J-JW analyzed the intracellular bacterial survival. C-LH, S-YH, Y-LC, and J-JW analyzed the cell response. C-LH, S-YH, S-LL, HO, TN, and J-JW did the images acquisition. C-LH, CC-N, P-XZ, and J-JW wrote the manuscript.

## Conflict of Interest

The authors declare that the research was conducted in the absence of any commercial or financial relationships that could be construed as a potential conflict of interest.
