# Supplementary material for: Nicotinamide Increases Intracellular NAD+ Content to Enhance Autophagy-Mediated Group A Streptococcal Clearance in Endothelial Cells
Source: Front Microbiol. 2020 Feb 11;11:117. doi: 10.3389/fmicb.2020.00117 (PMC7026195; doi:10.3389/fmicb.2020.00117)
Supplement: Supplementary file 1 [file Data_Sheet_1.pdf]

SUPPLEMENTARY DATA

FIGURE S1. The effects of nicotinamide on GAS growth *in vitro* and NADase activity. (A) The growth curve of GAS was determined in TSBY broth supplemented with nicotinamide (NAM). GAS was inoculated in TSBY broth containing different concentrations of NAM and the bacterial growth was measured by the optical density at 600 nm. **(B)** The NADase activity of GAS was measured in culture supernatants under NAM treatment. The culture supernatants were obtained from TSBY containing different concentration of NAM at 4 (mid-logarithmic phase) and 6 (late-logarithmic phase) of inoculation, and then incubated with β-NAD. The fluorescence intensity of β-NAD was measured by the spectrophotometer at an excitation wavelength of 360 nm. The NADase activity was expressed as relative percentage compared to medium alone.

FIGURE S2. Exogenous β-NAD and NADH treatment cannot increase intracellular NAD^+ content in GAS-infected endothelial cells. The intracellular NAD^+ content of endothelial HMEC-1 cells after exogenous NAD^+ substrate treatment. The cell lysates were extracted from GAS-infected HMEC-1 cells with/without exogenous β-NAD, NADH or nicotinamide (NAM), and analyzed by a

NAD$^+$/NADH quantification kit.

FIGURE S3. The autophagic LC3-II form cannot be induced in ATG9A-knockdown endothelial cells. (A) Cells were infected with GAS and the LC3 conversion was analyzed by Western blotting **(B)** Cells were infected with GAS under nicotinamide (NAM) treatment and the LC3 conversion was analyzed by Western blotting.
