# Supplementary figures and images for: Nicotinamide Increases Intracellular NAD+ Content to Enhance Autophagy-Mediated Group A Streptococcal Clearance in Endothelial Cells
Source: Front Microbiol. 2020 Feb 11;11:117. doi: 10.3389/fmicb.2020.00117 (PMC7026195; doi:10.3389/fmicb.2020.00117)

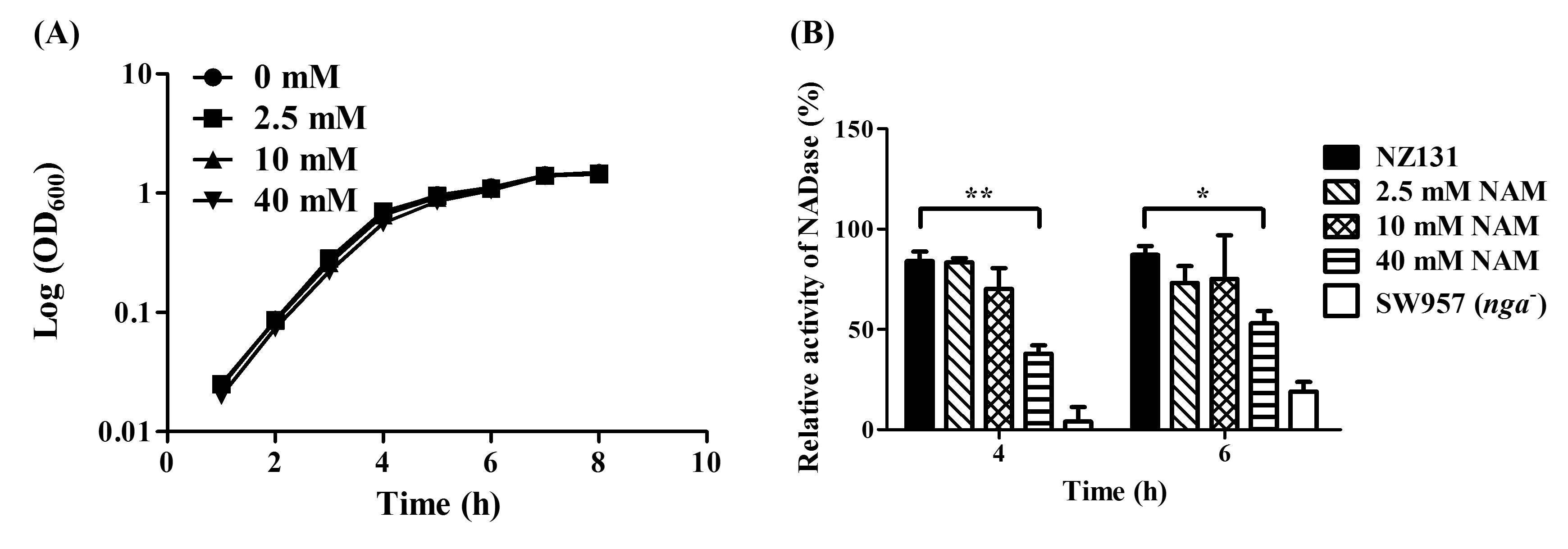

Supplement: Supplementary file 2 [file Image_1.TIF]

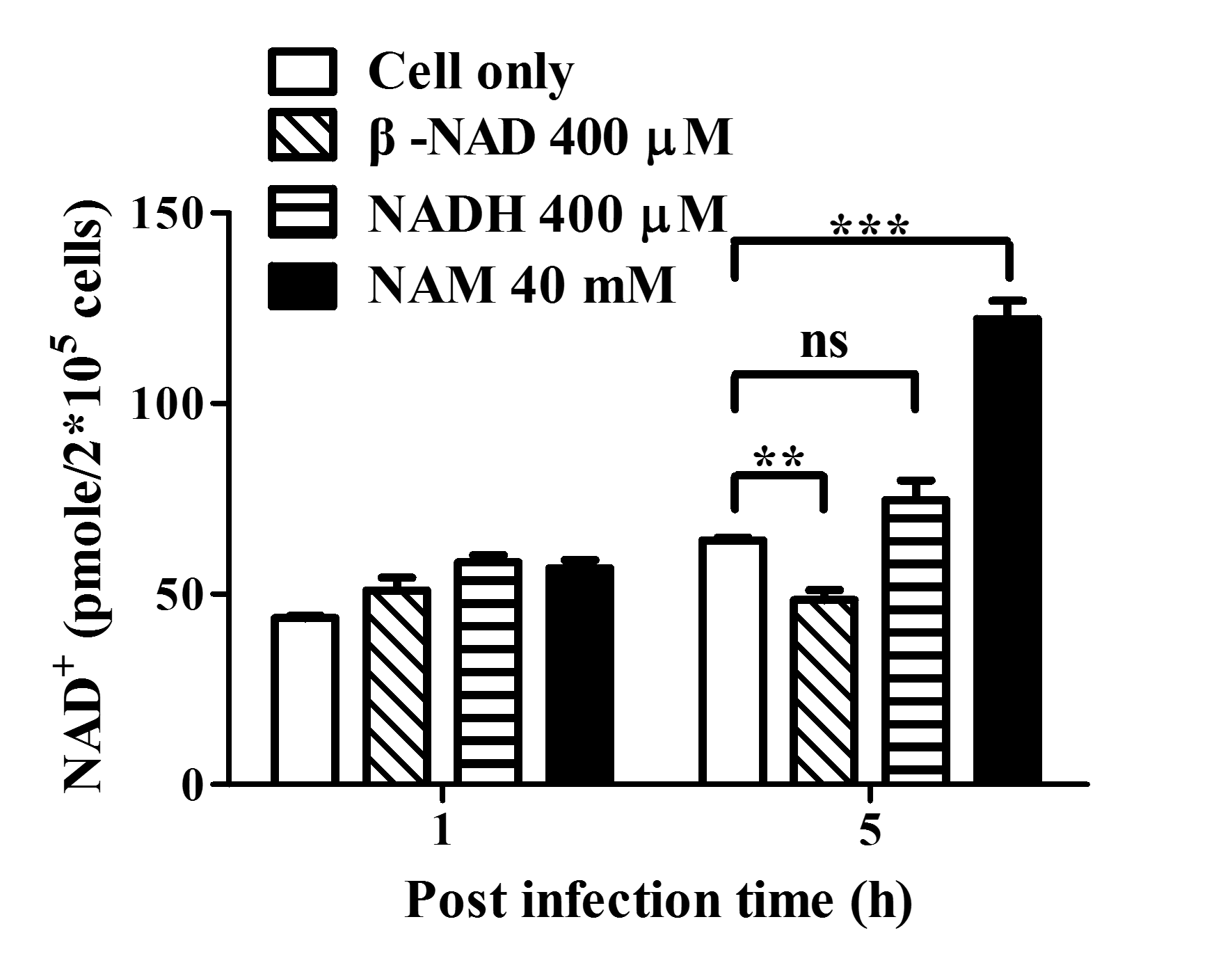

Supplement: Supplementary file 3 [file Image_2.TIF]

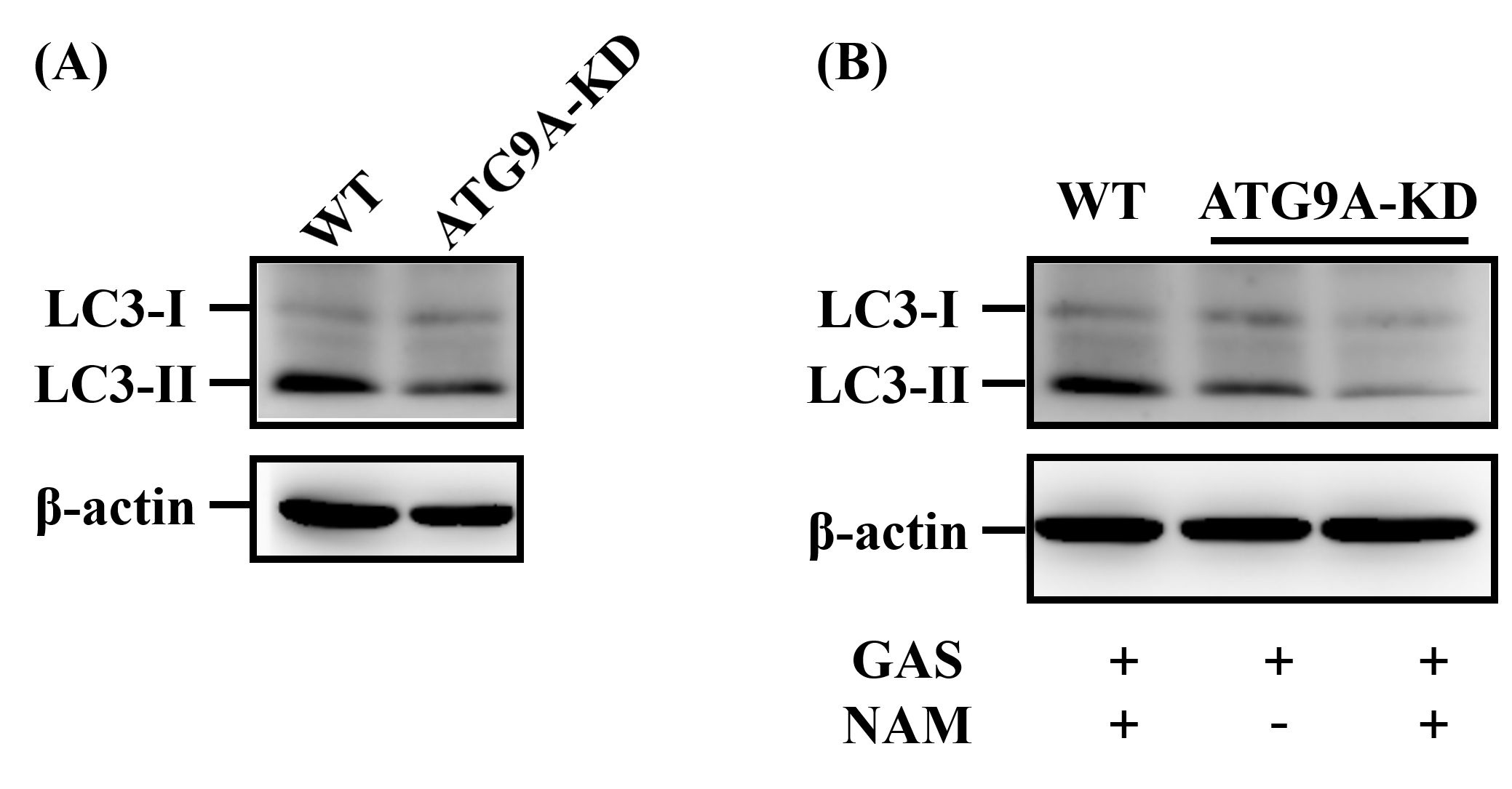

Supplement: Supplementary file 4 [file Image_3.TIF]
